# Tyrosine Kinase Inhibitor Independent Gene Expression Signature in CML Offers New Targets for LSPC Eradication Therapy

**DOI:** 10.3390/cancers14215253

**Published:** 2022-10-26

**Authors:** Eduardo Gómez-Castañeda, Lisa E. M. Hopcroft, Simon Rogers, Chinmay Munje, Joana Bittencourt-Silvestre, Mhairi Copland, David Vetrie, Tessa Holyoake, Heather G. Jørgensen

**Affiliations:** 1 Paul O’Gorman Leukaemia Research Centre, Institute of Cancer Sciences, College of Medical, Veterinary and Life Sciences, University of Glasgow, 21 Shelley Road, Glasgow G12 0ZD, UK; 2 School of Computing Science, College of Science and Engineering, University of Glasgow, 18 Lilybank Gardens, Glasgow G12 8RZ, UK; 3 Wolfson Wohl Translational Cancer Research Centre, Institute of Cancer Science, College of Medical, Veterinary and Life Sciences, University of Glasgow, Glasgow G61 1QH, UK

**Keywords:** tyrosine kinase inhibitors, cancer stem cells, treatment persistence, target discovery, CML, BCR-ABL1, BCR-ABL1 independence

## Abstract

**Simple Summary:**

Chronic myeloid leukaemia (CML) is initiated by a group of cancer cells called leukaemia stem cells (LSC). These LSC can survive current tyrosine kinase inhibitor (TKI) treatments and, upon treatment withdrawal, are able to re-initiate the disease. Thus, eradicating the LSC would likely cure CML. In this study, we have identified a number of genes whose expression is different between LSC and their healthy counterparts (haematopoietic stem cells) but are not affected by TKI treatment. We hypothesised that these genes may be potential therapeutic targets against LSC and used two different drugs, gemtuzumab–ozogamicin and cyclosporine A, to treat CML in vitro. We found that both drugs have a stronger effect on CML cells than on healthy cells. Therefore, we propose that the list of genes we identified could represent a novel source of therapeutic targets against CML.

**Abstract:**

Tyrosine kinase inhibitors (TKI) have revolutionised the treatment of CML. However, TKI do not eliminate the leukaemia stem cells (LSC), which can re-initiate the disease. Thus, finding new therapeutic targets in CML LSC is key to finding a curative treatment. Using microarray datasets, we defined a list of 227 genes that were differentially expressed in CML LSC compared to the healthy controls but were not affected by TKI in vitro. Two of them, *CD33* and *PPIF*, are targeted by gemtuzumab–ozogamicin and cyclosporin A, respectively. We treated CML and the control CD34^+^ cells with either drug with or without imatinib to investigate the therapeutic potential of the TKI-independent gene expression programme. Cyclosporine A, in combination with imatinib, reduced the number of CML CFC compared with non-CML controls, but only at supra-therapeutic concentrations. Gemtuzumab–ozogamicin showed an EC_50_ of 146 ng/mL, below the plasma peak concentration of 630 ng/mL observed in the AML patients and below the EC_50_ of 3247 ng/mL observed in the non-CML cells. Interestingly, gemtuzumab–ozogamicin seems to promote cell cycle progression in CML CD34^+^ cells and demonstrated activation of the RUNX1 pathway in an RNAseq experiment. This suggests that targeting the TKI-independent genes in CML LSC could be exploited for the development of new therapies in CML.

## 1. Introduction

The introduction of tyrosine kinase inhibitors (TKIs) into clinical practice has greatly improved the survival prognosis of patients diagnosed with chronic myeloid leukaemia (CML) [1]. However, TKIs are unable to eradicate the disease, as leukaemic stem cells (LSCs) persist despite treatment [2,3,4]. This means that most patients need life-long therapy with its associated side effects [5], which is not only a financial and psychological burden but upon which the risks of resistance, disease recrudescence and progression are contingent.

TKI treatment, although effective at eradicating CML cells, has been shown to increase the proportion of quiescent LSCs [2,3,4,6], partially because of the potential for the induction of quiescence and self-renewal gene expression [7,8]. Moreover, the inhibition of BCR-ABL1 TK activity alone is not enough to eradicate CML progenitor cells [9] or LSCs [10]. Thus, it is possible that CML LSCs’ aberrant signalling is not entirely driven by BCR-ABL1 TK. For example, it has been reported that the expression of *MIR10A*, a miRNA that is downregulated in CML cells, is not dependent on BCR-ABL1 TK, but its downregulation promotes proliferation and cell growth in CML cells [11]. As TKI monotherapy is insufficient to eradicate CML LSCs, this suggests that several genes independent of BCR-ABL1 TK may be supporting these BCR-ABL1 TK-independent cells.

This led to the hypothesis that there is a gene expression programme in CML LSCs independent of the BCR-ABL1 TK activity that is required for their survival. Previous reports have investigated the presence of deregulated genes in CML cells that are not corrected by TKI in therapy-resistant cells. For example, the β-catenin protein levels of CML CD34^+^ cells from patients resistant to more than one TKI are unaffected by TKI treatment in vitro [12]. Additionally, MYC and p53 pathways are deregulated in CML LSC regardless of the responsiveness to TKI treatment and, upon investigation, were shown not to be targeted by TKI [13].

**Figure 1:**
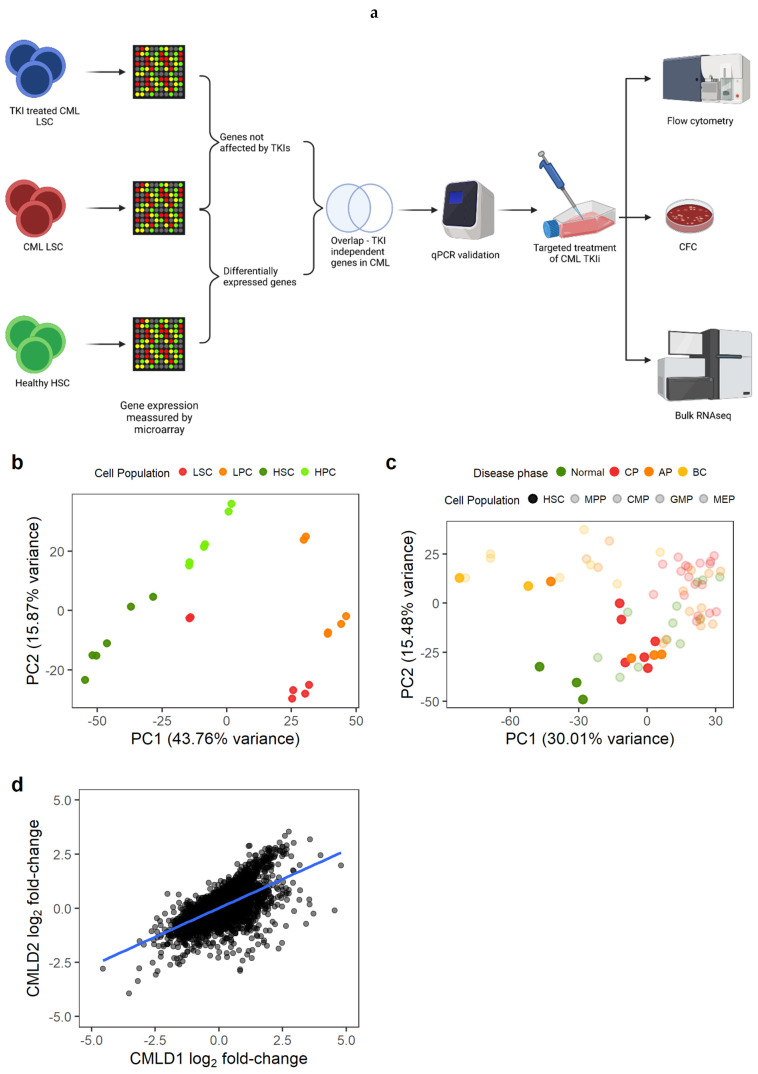
Gene expression changes in CML LSC are consistent across datasets. (**a**) Experimental design. We identified genes differentially expressed in CML LSC but not affected by TKI treatment (expression measured by microarrays) and selected two targets for targeted therapy against CML LSC in combination with TKI. The effect of such therapies was studied by flow cytometry (cell cycle), CFC and RNAseq. (**b**) PCA plot of CMLD1. Each dot represents a microarray (including technical duplicates). Technical duplicates were close to each other. The samples clustered following two axes: CML/normal and stem/progenitor cells. (**c**) PCA plot of CMLD2. Each dot represents one microarray (no technical replicates in this dataset). CML-CP LSC co-localise with normal progenitor cells, suggesting a progenitor-like phenotype in CML-CP LSC. (**d**) Gene expression log_2_ fold-changes between CML LSC and normal HSC were significantly correlated between CMLD1 and CMLD2 (Pearson’s R = 0.63). Each dot represents an individual gene. Figure 1a was created with BioRender.com.

Based on the current evidence, we hypothesised that treatment naïve CML CD34^+^ cells possess a gene expression programme independent of the BCR-ABL1 TK, which allows them to persist following TKI treatment and that the components of this programme could be targeted to eradicate the leukaemic clone. By investigating CML CD34^+^ cells treated with TKI and untreated, as well as CML LSC and normal haemopoietic stem cells (HSC), we identified a list of 227 genes that are TKI-independent (TKI-independent) and potentially BCR-ABL1 TK-independent. This list includes *CD33*, a myeloid cell-surface marker; *PPIF*, a cell-death regulator; and *ERG*, a transcriptional factor involved in HSC development and maintenance. We used gemtuzumab–ozogamicin (GO), an anti-CD33 conjugated monoclonal antibody approved by the Food and Drug Administration (FDA) and the European Medicines Agency (EMA) for the treatment of acute myeloid leukaemia [14] and cyclosporin A (CsA), a blocker of PPIF activity [15] for targeting CML CD34^+^ cells under physiological conditions in vitro (Figure 1a). *ERG* downregulation mimics *MYC* overexpression, and it has been shown that bromodomain and extraterminal (BET) inhibitors restore normal expression in ERG-deficient mice [16]. BETi have already been successfully shown to eliminate CML LSC in our lab [13].

## 2. Material and Methods

### 2.1. Code Availability

The code used for the data analysis can be found in GitHub EduardoGCCM/Gomez-Castaneda2022_TKIi.

### 2.2. Primary Patient Material

All of the samples were collected after obtaining written informed consent from the patients. The project had ethical approval from the West of Scotland Research Ethics Committee 4 (REC reference: 15-WS-0077). The cells were processed for cryopreservation from the peripheral blood or leukapheresis from patients diagnosed with CML and other haematological malignancies or healthy donors (‘non-CML’ including allogeneic haematopoietic stem cell donors). Patients’ age, biological sex and response to imatinib (IM) are summarised in Tables S1 (CML) and S2 (non-CML). Cells were enriched for CD34^+^ using magnetic-activated cell sorting (MACS) or CliniMACS. 

### 2.3. Microarray Analysis

Data analysis was performed using R 4.1.1 running under MS Windows 10 21H1 unless otherwise stated. 

The microarray intensities were normalised using the RMA algorithm [17,18,19] implemented in the *oligo* package [20]. The microarray chip annotations were summarised at the transcript level using the *getNetAffx* function. Microarray differential gene expression was calculated using *limma* [21,22]. The statistically significant probe sets were annotated to gene names (HGNC symbols) with *biomaRt* [23] connecting to the Ensembl release in August 2020 [24]. 

The microarray datasets used for the comparison of the CML and non-CML stem cells (LSC/HSC) were CMLD1 (Array Express accession number E-MTAB-2581) [13] and CMLD2 (GEO accession number GSE47927) [25]. CMLD1 comprises of LSC/HSC (CD34^+^CD38^−^) and progenitor cells (CD34^+^CD38^+^) from three CML patients and three non-CML patients. The samples from CMLD1 were run in technical duplicates. Only the LSC/HSC were used for the differential expression analysis, and the technical replicates were accounted using the *duplicateCorrelation* function from *limma*. CMLD2 comprises of a collection of cell populations of varying maturity—LSC/HSC (CD34^+^CD38^−^CD90^+^), MPP (CD34^+^CD38^+^CD90^−^), CMP (CD34^+^CD38^+^CD123^+^CD45RA^+^), GMP (CD34^+^CD38^+^CD123^+^CD45RA^low^) and MEP (CD34^+^CD38^+^CD123^−^CD45RA^−^)—sorted from healthy donors (*n* = 3), chronic phase (*n* = 6), accelerated phase (*n* = 4) and myeloid blast crisis CML patients (*n* = 2). Only LSC/HSC from the healthy and CML chronic phases were used for the analysis. A q-value smaller than 0.1 (Benjamini–Hochberg correction [26]) was considered statistically significant. 

The comparison between the TKI-treated and untreated cells was made by comparing the samples in TKID1 (Array Express accession number E-MTAB-2594) [27]. The gene expression was measured at baseline (0 h) and after eight hours of treatment (8 h) for CD34^+^CD38^-^ and after 7 days of treatment (7 d) for CD34^+^ cells. The cells were treated with the clinically achievable drug concentrations of 5μM imatinib (IM), 150 nM dasatinib or 5 μM nilotinib (one replicate each); a second dose of the drug at the same concentration was applied during the 4th day of treatment. The cells were sorted again for the live cells after 7 d of treatment before RNA extraction. Statistical significance for no change was assessed using an equivalence test [28].

All of the microarrays analysed were performed using Affymetrix HuGe 1.0 ST chips, and the RNA was extracted using RNeasy Micro Kit (Qiagen) when the number of isolated cells was less than 5 × 10^5^, and RNeasy Mini Kit (Qiagen) when the number of isolated cells was between 5 × 10^5^ and 1 × 10^7^.

### 2.4. RNA Sequencing

The RNA was extracted using the Arcturus PicoPure kit for patients CML423 and CML460 and using the RNAeasy Micro kit for patient CML441. The RNA was reverse-transcribed using the SMART-Seq v4 Ultra Low Input RNA Kit for Sequencing (Takara, Saint-Germain-en-Laye) by Glasgow Polyomics. The cDNA library preparation was performed using the Nextera library preparation kit by Glasgow Polyomics. The RNA sequencing was performed using an Illumina HiSeq 4000 sequencer. The dataset can be found in GEO (GSE198576).

### 2.5. Bulk RNA-Seq Analysis

The RNAseq quality of sequencing was assessed using *FastQC* [29], and the sequences were trimmed using *cutadapt* [30] with default parameters on pair-end mode removing the last base of each read, filtering out reads shorter than 20 bp. The release v31 of the GRCh38.p12 human genome was used as a reference, and the reads were aligned to the genome using *STAR* [31]. A count matrix was generated using *featureCounts* (Liao et al., 2013). Differential gene expression was calculated using *edgeR* [32]. A q-value smaller than 0.1 (Benjamini–Hochberg correction [26]) was considered statistically significant.

### 2.6. Validation of the TKI-Independent Signature

The cells were seeded in serum-free media (SFM) in a 6-well plate and grouped as no drug control (NDC), or IM-treated, as previously described for the TKID dataset [27]. IM was added at a final concentration of 5 μM. The cells were cultured at 37 °C and 5% CO_2_ for 7 days. IM was added again on day 4 without washing the cells. On day 7, the cells were sorted for viable cells (DAPI^−^) by flow cytometry using a BD FACS Aria with Diva software and used for RNA extraction. The RNA was extracted using the RNeasy Micro Kit (Qiagen) when the number of isolated cells was less than 5 × 10^5^ and the RNeasy Mini Kit (Qiagen) when the number of isolated cells was between 5 × 10^5^ and 1 × 10^7^.

### 2.7. qPCR Analysis

qPCR was performed using a Fluidigm 48.48 PCR chip following the manufacturers protocol.

The cDNA of each sample was pre-amplified for 18 cycles using the PCR multiplex PCR kit (Qiagen). Each reaction contained a pool of all the primers of interest at 50 nM each and a maximum of 12.5 ng of cDNA (some samples yielded very low concentrations of RNA, and higher concentrations were not possible). The polymerase was activated at 95 °C for 15 min, and each cycle comprised 30 s of denaturation at 94 °C, 90 s of annealing at 60 °C and 60 s of extension at 72 °C. A final extension of 30 min at 72 °C was performed. The samples were treated with 0.5 U/μL of exonuclease I (New England Biolabs, Ipswich, MA, USA) for 30 min at 37 °C. The enzyme was inactivated at 80 °C for 15 min. The samples were diluted to 1:5 and stored. 

The 48.48 chip was primed with control line fluid in the IFC controller MX, and each of the primer wells was filled with a 5 μL solution containing 1X assay-loading reagent, The DNA suspension buffer and 5 μM of each of the primers of the pair assigned to the well. Each sample was loaded with 5 μL of 1X SsoFast™ EvaGreen Supermix with low ROX (Bio-Rad), 1X DNA-binding dye sample-loading reagent (Fluidigm) and 45% *v*/*v* of the pre-amplified cDNA assigned to the well. The reaction in the Biomark activated the enzyme at 95 °C for 1 min and performed 30 cycles of denaturation at 96 °C for 5 s and annealing and extension at 60 °C for 20 s. A melting curve was generated at the end of the qPCR for every reaction.

The relative expression of the test genes was calculated by subtracting the mean of the Ct values of the reference genes (*ENOX2*, *GAPDH*, *RNF20*, and *TYW1*) from the Ct value of the test gene within each sample (ΔCt). These ΔCt values were used as normalised gene expression values, and the differential gene expression was calculated using *limma* (Ritchie et al., 2015). The genes were considered to be differentially expressed when the BH-adjusted *p*-value was lower than 0.1. The confidence interval of the ΔΔCt (log2 fold change) was also calculated by *limma*. A gene was considered non-changing when its median ΔΔCt was within the interval of −0.5 to 0.5.

### 2.8. Drug Response Experiments

The CD34^+^ primary cells were resuspended at a density of 2 × 10^5^ cells/mL in SFM + physiological growth factors (0.2 ng/mL SCF, 1 ng/mL G-CSF, 0.2 ng/mL GM-CSF, 1 ng/mL IL6, 0.05 ng/mL LIF, 0.2 ng/mL MIP1α) in the presence or absence of 2 µM IM and/or different concentrations of either GO (10, 30, 100, 300 and 1000 ng/mL) or CsA (0.3, 1, 3, 5, 10 and 30 μM) and cultured at 37 °C and 5% CO_2_. The treatment was delivered in three different regimens:

- 72 h of GO or CsA with or without IM.
- 72 h IM (or no drug) followed by 72 h of either GO or CsA.
- 72 h of either GO or CsA followed by 72 h of IM (or no drug).

Following 72 h, the cells were washed in PBS and centrifuged for 10 min at 300 g three times in order to washout the first drug. After the treatment, the viable cells were manually counted using trypan blue dye exclusion using a haemocytometer and were then used for the downstream experiments.

### 2.9. Colony Forming Cell Assays

For the colony-forming cell (CFC) assay, 3000 cells were mixed with 3 mL of Methocult^®^ H4034 (Stem Cell Technologies). This mix was then split evenly between two 35 mm^2^ plates covering the entire surface of the plates. All of the plates from each sample were placed inside a 23.5 cm^2^ plate, and 2 plates containing just water were added to avoid the Methocult drying. The cells were cultured for no less than 9 days at 37 °C and 5% CO_2_ before analysis.

### 2.10. Cell Cycle Analysis

The cells were fixed using 80% ethanol at −20 °C and stored at 4 °C before analysis. The DNA was stained using DRAQ7 dye and analysed on a BD FACS Aria (BD Biosciences) with Diva software. Data analysis was performed with FlowJo 10.7.2.

### 2.11. Other Data Analysis

Unless otherwise stated, the data analysis was performed using R 4.1.1 on Windows 10 21H1. A q-value smaller than 0.1 was considered statistically significant (Benjamini–Hochberg correction [26]. The drug–response curves were calculated using the *drc* package [33].

## 3. Results

### 3.1. CML LSC Possess a TKI-Independent Gene Expression Signature

We first identified the genes that were differentially expressed (DE) between CML LSC and non-CML HSC (Figure 1a). We used two previously generated microarray datasets comparing non-CML to treatment naïve CML cells. As described in the methods section, CMLD1 [13] and CMLD2 [25] were generated using the same Affymetrix chips and the most primitive populations (HSC/LSC) were sorted for CD34^+^CD38^−^ and CD34^+^CD38^−^CD90^+^, respectively. The first two principal components (PC) of CMLD1 showed a clear separation between CML and non-CML, as well as between stem (CD34^+^CD38^−^) and progenitor cells (CD34^+^CD38^+^), as expected (Figure 1b). The first two PC of CMLD2 showed non-CML HSC in a distinct position, while chronic phase (CP) CML and most accelerated phase (AP) CML LSC clustered with non-CML progenitors, suggesting that CP and AP CML LSC have a progenitor-like molecular phenotype (Figure 1c), as previously reported [34]. The blast crisis (BC) LSC and one AP LSC sample clustered together, as previously shown using scRNAseq [6]. When comparing CML LSC against non-CML HSC in CMLD1, we identified 4505 DE genes, while the same comparison in CMLD2 identified 2344 DE genes. In order to investigate only genes with a high confidence of being DE in CML LSC compared with non-CML HSC, we used the 1497 genes that were DE in both datasets. This overlap (or higher) was very unlikely to happen by chance (*p* < 0.001, hypergeometric distribution). Additionally, we investigated if the global changes in the transcriptome were conserved across datasets (Figure 1d). We found significant positive correlation (*p* < 0.001; R_1d_ = 0.63; Pearson’s correlation).

**Figure 2:**
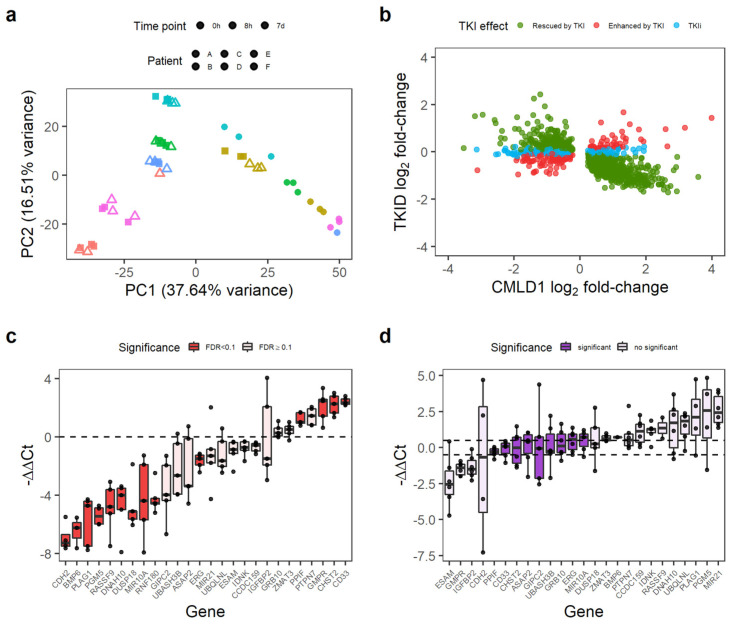
Some genes differentially expressed in CML LSC are not affected by TKI treatment. (**a**) PCA of the TKID dataset. Interpatient variability and treatment (7 d vs. 0 h/8 h) seem to have a major contribution to the variance of the dataset. Each point represents one microarray. (**b**) Correlation of gene expression log2 fold-changes between TKID and CMLD1. Although most genes show a negative correlation after 7 d of TKI treatment compared with CML vs. Normal (normal expression “rescued by TKI”), a minority of genes were not affected by TKI treatment (TKI independent or TKIi), and some genes were further deregulated into the same direction than in CML vs. Normal (“enhanced by TKI”). (**c**) Gene expression differences between CML-CP CD34^+^ cells and normal CD34^+^ cells as measured by qPCR. Each dot represents the relative expression of one CML sample compared with the mean expression of the normal samples for each gene. Differential gene expression was calculated with *limma*, and an FDR < 0.1 was considered significant. (**d**) Gene expression differences between CML-CP CD34^+^ cells treated with 5 µM IM (7 d) and NDC (7 d). Relative gene expression was calculated per patient (paired-analysis), and a mean log2 fold-change (or ΔΔCt) between −0.5 and 0.5 was considered significant no change. Each dot represents a patient.

Then, we identified the genes not affected by the TKI treatment using the TKID dataset. TKID was generated with the same Affymetrix chips as CMLD1 and CMLD2 and contained the gene expression data of the treatment naïve chronic phase CML samples at baseline (CD34^+^CD38^−^ cells) and after 7 days in vitro TKI treatment (7 d, CD34^+^ cells). The PCA suggests a strong patient effect (patients indicated by colour), with the cells treated with TKI for 7 d being different from the cells at baseline or the cells after early exposure (Figure 2a). As we were trying to identify the genes not affected by the TKI treatment (as a proxy for the genes independent from BCR-ABL1 TK), we decided to assess the TKI-independent (TKI-independent) genes using an equivalence test, as previously described [28]. As equivalence tests require a threshold or margin of equivalence to be set by the research team, we decided to use the expected technical variation. Thus, we calculated the log_2_ fold-changes of the technical replicates present in CMLD1 and TKID in order to identify the expected differences between the biologically identical samples. As a *p*-value threshold of 0.05 is commonly used as a reference for significant change, we selected the percentiles 2.5 and 97.5, as the threshold for change. These percentiles were −0.486 and 0.487 log_2_ fold-change, which we rounded to −0.5 and 0.5 for the remaining calculations. Thus, any log_2_ fold-change whose 95% confidence interval crossed zero and was contained within −0.5 to 0.5 was considered non-changing. We identified 4913 non-changing genes after 7 d of TKI treatment in vitro.

Overall, 74.2% (1111/1497) of the consistently DE genes between CML and non-CML were rescued by TKI (i.e., the effect of TKI treatment was opposite to that of CML transformation). However, the expression of 159 genes was further de-regulated after TKI treatment (e.g., genes already upregulated in CML were further upregulated), and 227 genes were not affected by the TKI treatment (Figure 2b). We termed these latter genes TKI-independent genes and focused on them as potential therapeutic targets for combination treatments with TKIs. The 227 TKI-independent genes can be found in Table S3.

We selected a group of 26 genes, which had an absolute log_2_ fold-change of at least 0.7 in CMLD1 (only *CHST2* and *IDNK* had smaller fold-changes) and had biological relevance for validation by qPCR in an independent cohort of five CML-CP patients and two healthy donors. The qPCR experiment confirmed that of the 26 genes, 14 were DE between CML and non-CML (Figure 2c) and nine were not affected by IM (Figure 2d). *PPIF*, *ERG*, *MIR10A*, *CD33* and *CHST2* were TKI-independent in the validation experiment. As PPIF and CD33 can be targeted by existing drugs (CsA and GO, respectively), we focused on these two genes for targeting the TKI-independent signature in CML LSCs. 

### 3.2. Anti-CD33 Therapeutic Antibody Can Be Used to Specifically Eradicate CML CD34^+^ Cells

GO is a conjugated monoclonal antibody that releases the toxic antibiotic calicheamicin intracellularly after it binds to its target, CD33. Calicheamicin produces double-strand breaks in the DNA, leading to cell death. Currently, GO is approved for the treatment of AML by both the FDA and the EMA [35]. Thus, we consider it a potential candidate for re-purposing. Furthermore, a previous study found GO to be effective at eradicating CML mononuclear cells [36]. We also confirmed by flow cytometry that CD33 was expressed on CML CD34^+^ cells’ surface (Figure S1) and that it being on the cell surface did not significantly change after IM treatment (Figure S2).

We treated the CML and non-CML CD34^+^ cells using three different regimens in vitro to mimic potential clinical scenarios when used in combination with IM: GO or GO + 2µM IM for 72 h treatment (GO_72_); 72 h IM or NDC followed by 72 h GO treatment (GO_IMGO_); or, 72 h GO followed by 72 h IM or NDC (GO_GOIM_).

**Figure 3:**
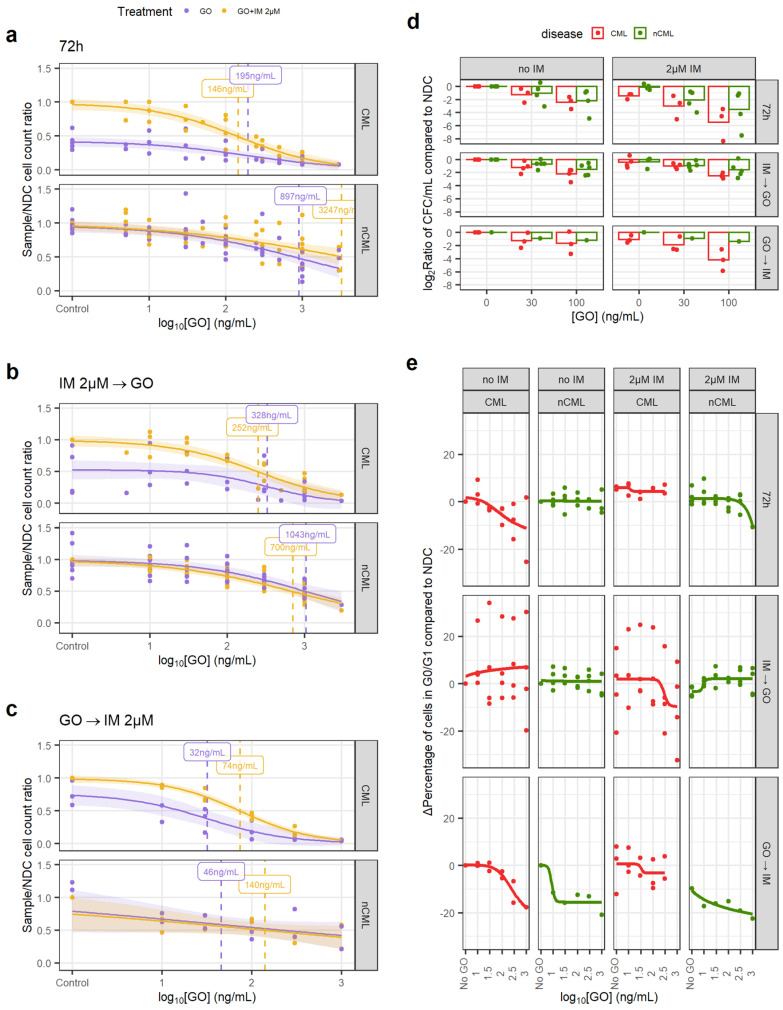
GO is effective at eradicating CML CD34^+^ cells. (**a**) Dose–response curves for the GO_72_ treatment regimen. (**b**) Dose–response curve for the GO_IMGO_ treatment regimen. (**c**) Dose–response curve for the GO_GOIM_ treatment regimen. (**d**) The reduction in the number of CFC was calculated dividing the total number of CFC in the original liquid culture after treatment and dividing this number by the number of CFC in the original liquid culture of the corresponding NDC (patient-matched). These ratios were log_2_-transformed. A linear model fitted using GO_72_ and GO_IMGO_ data showed that GO significantly reduces CFC numbers in CML samples more than in nCML samples. (**e**) Change in the percentage of quiescent (G_0_/G_1_) cells after treatment at different concentrations and regimens of GO. Cell cycle was assessed using DRAQ7 DNA staining (flow cytometry). (All) The shadowed area represents the 95% confidence interval, and the dashed line the EC_50_ (value given in the figure). Each dot represents an individual patient sample (across different concentrations). Curves and EC_50_ were calculated using R’s *drc* package.

Using the GO_72_ regimen, the CML CD34^+^ cells had an EC_50_ of 146 ng/mL when treated with GO alone but 195 ng/mL when combined with IM (Figure 3a). These EC_50_s were not statistically significantly different from each other and, thus, the effect of GO and IM can be considered independent. These concentrations are achievable in vivo with the current recommended dosing for the treatment of AML (plasma C_max_ of 630 ng/mL) [35]. Additionally, GO on non-CML controls had an EC_50_ > 3000 ng/mL that decreased to 897 ng/mL in combination with IM (Figure 3a), both being significantly higher than respective EC_50_ for CML cells. These results show that GO has a wide therapeutic window and that non-CML EC_50_ remains higher than clinically achievable plasma peak concentrations in patients under the current dosing recommendation for GO for AML.

The GO_IMGO_ regimen showed a similar trend, with both GO alone and in combination with IM having significantly lower EC_50_ in CML than in non-CML and within in vivo achievable concentrations (Figure 3b). EC_50_ in non-CML remained higher than the peak plasma concentrations in the patients.

The GO_GOIM_ regimen showed an overall reduction in EC_50_ for all conditions. Although the number of samples tested with this regimen is lower than the other two, it might suggest that GO cytotoxic effect may persist after the drug has been removed and washed out from the culture (Figure 3c).

We observed similar results after Annexin V staining in all three treatment regimens (Figures S3–S5).

In order to investigate the effect of GO on stem and progenitor cells (SPC), CFC counts and cell cycle analysis were performed. When comparing the treated/NDC log_2_ CFC ratios, the CML cells consistently showed a greater decrease in CFCs than non-CML in response to the drug, which was significant in a linear model (1.46-fold bigger decrease than non-CML, *p* = 0.024; Figure 3d). GO_GOIM_ was not included in the linear model due to the imbalance of CML/non-CML sample number. This suggests that CML SPC are more susceptible to GO than non-CML SPC.

IM is known for inducing quiescence in SPC, protecting CML LSC from eradication. Thus, combining IM with a drug that pushes CML LSC towards the cell cycle may increase their vulnerability to IM. Using DNA staining, we observed a decrease in the number of CML cells in either G_0_ or G_1_ when treated with either GO_72_ or GO_GOIM_ regimens but not with GO_IMGO_. The IM-treated CML cells showed an increase in cells in the G_0_/G_1_ phases that was only modestly decreased after the GO treatment (Figure 3e, Figures S6 and S7). Under the GO_IMGO_ regimen, this was not observed, but it could be due to the increased cell concentration in the culture after 72 h without treatment, which could affect either the growth potential of the cells or reduce the drug-to-target ratio or other culture artifacts. The effect of GO on the cell cycle stage of non-CML cells was minimal, especially at concentrations equal to, or lower than, CML EC_50_.

### 3.3. CsA Targets CML SPC When in Combination with IM

CsA is an immunosuppressant drug that has been used for the treatment of transplant rejection and graft versus host disease (GvHD) since the 1980s. It is known to inhibit the activation of cytotoxic T-cells. However, it has several side effects due to its broad mechanism of action [37]. Interestingly, CsA has also been proposed as a treatment to prevent necrosis in ischemic events due to its ability to inhibit the activity of PPIF (also known as cyclophilin D) and its interaction with p53 [15]. Thus, we tested whether inhibiting PPIF, which is upregulated in CML LSC compared to non-CML HSC, would have any benefit in the treatment of CML.

Similar to the experiments using GO, we treated the CML and non-CML CD34^+^ cells using three different regimens in vitro, to mimic potential clinical scenarios when used in combination with IM: CsA or CsA + 2 µM IM 72 h treatment (CsA_72_); 72 h IM or NDC followed by 72 h CsA treatment (CsA_IMCA_); or, 72 h CsA followed by 72 h IM or NDC (CsA_CAIM_).

**Figure 4:**
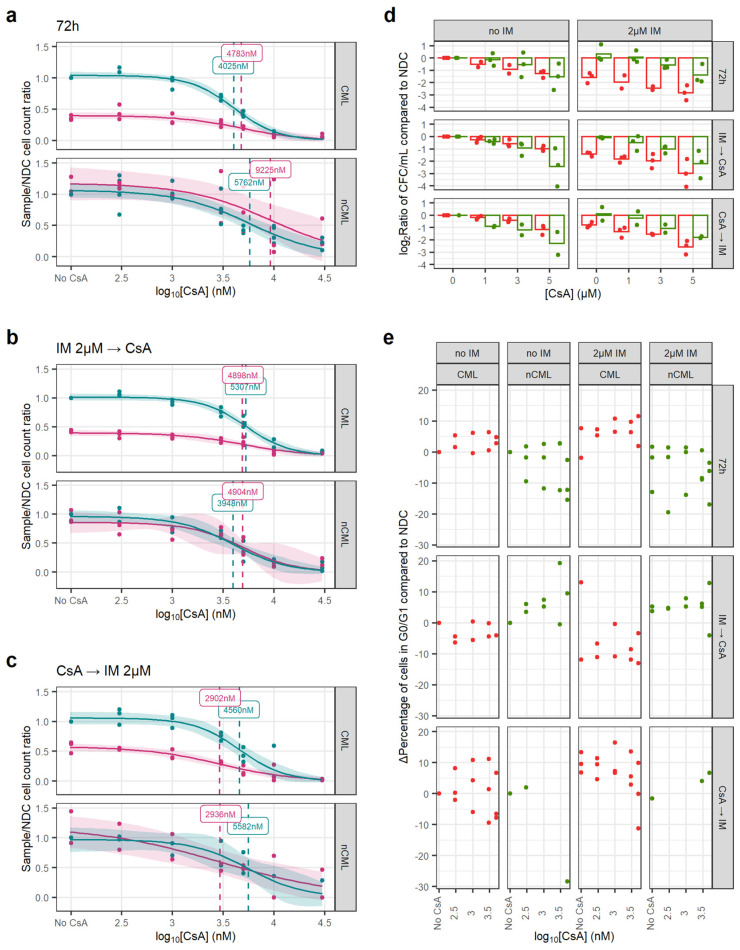
CsA eradicates CML CFC in combination with IM. (**a**) Dose–response curves for the CsA_72_ treatment regimen. (**b**) Dose–response curve for the CsA_IMCA_ treatment regimen. (**c**) Dose–response curve for the CsA_CAIM_ treatment regimen. (**d**) The reduction in the number of CFC was calculated dividing the total number of CFC in the original liquid culture after treatment and dividing this number by the number of CFC in the original liquid culture of the corresponding NDC (patient-matched). These ratios were log_2_-transformed. Using all treatment regimens, a linear model showed that CsA significantly decreases more the number of nCML CFC than CML CFC in the absence of IM. However, in combination with IM, CsA significantly reduces the number of CFC in CML than in nCML. (**e**) Change in the percentage of quiescent (G_0_/G_1_) cells after treatment at different concentrations and regimens of CsA. Cell cycle was assessed using DRAQ7 DNA staining (flow cytometry). (All) The shadowed area represents the 95% confidence interval, and the dashed line the EC_50_ (value given in the figure). Each dot represents an individual patient sample (across different concentrations). Curves and EC_50_ were calculated using R’s *drc* package.

In all three regimens, the EC_50_ of CsA, either alone or in combination with IM, was similar, suggesting an effect independent of IM. However, CsA’s EC_50_ for CML and non-CML were similar at 3000–5000 nM and over the 1000 nM peak concentration usually achieved in clinical practice (Figure 4a–c) [38]. 

Similarly, CsA alone has a greater effect on the number of non-CML CFCs than on CML (*p* = 0.017). However, this trend is reversed when the cells have also been treated with IM, independently of the treatment regimen (*p* < 0.001) (Figure 4d). This potentially means that CsA synergies with IM when targeting CML SPC.

In contrast with GO, CsA did not induce cell cycle progression and regimens/conditions showed increased cell cycle arrest with increasing concentrations of CsA, with or without IM (Figure 4e). 

### 3.4. Transcriptomic Changes after GO, CsA and IM Treatments

**Figure 5:**
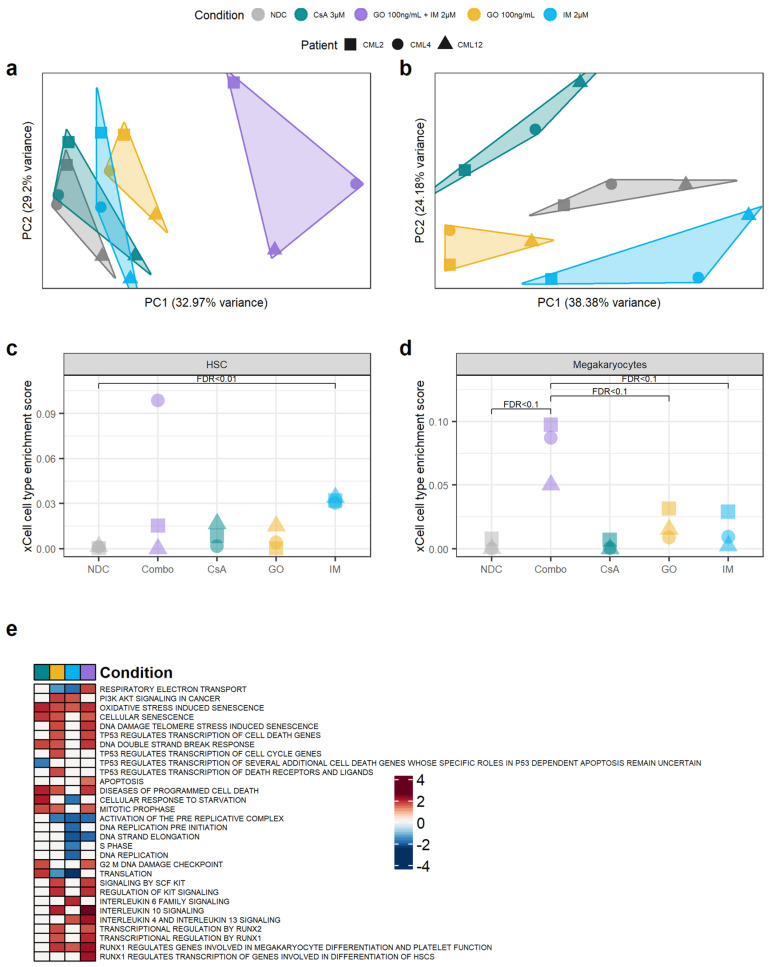
Targeting the TKIi signature alters the transcriptome of CML CD34^+^ cells. (**a**) PCA plot of all the samples sequenced. The transcriptomic changes caused by the GO + IM combination explain most of the variability of the dataset. (**b**) PCA plot after removing the GO + IM combination samples. This allows for a better understanding of the differences between the single treatments, which seem to be independent of each other (each treatment is projected in a different direction from the NDC). (**c**) Comparison of the HSC enrichment score generated by xCell for each sample. IM-treated samples were significantly enriched by HSC compared to the NDC (FDR < 0.01). (**d**) Comparison of the megakaryocyte enrichment score generated by xCell for each sample. The GO + IM was borderline significantly enriched on megakaryocytes compared either to the NDC or to GO/IM single treatment (FDR < 0.1). (**e**) Shortlist of the Reactome pathways significantly enriched in any of the conditions by GSEA. White squares represent non-significant pathways (independently of the enrichment score). Blue squares represent downregulated pathways, while red squares represent upregulated pathways. Full list of pathways can be found in Figure S8. (all) Each shape represents an individual patient, while each colour represents an individual treatment.

Our results suggest that GO, and to some extent CsA, may be effective in eradicating CML CD34^+^ cells. To investigate the mechanisms of action by which GO and CsA, as well as IM, eradicate CML, we performed bulk RNAseq from the cells treated with 100 ng/mL of GO, 100 ng/mL of GO + 2 µM IM, 2 µM of IM and 3 µM of CsA for either GO_72_ or CsA_72_ treatment regimens. As expected, the GO + IM combination treatment had the biggest impact on gene expression compared to the other conditions (Figure 5a). To increase the sensitivity for gene expression changes in the other treatments, differential gene expression from the single-agent treatments was performed on the data normalised in the absence of the GO + IM combination. Removing the GO + IM combination allowed us to observe different clusters of samples according to their single-agent treatment (Figure 5b). Interestingly, none of the single-agent clusters overlapped, and the NDC is projected to the centre of the plot when using the first two PCs. This may suggest that all three single agents affect different transcriptional pathways.

Compared to the NDC, the GO + IM combination had 3789 DE genes, GO single-agent 1268 DE genes, CsA single-agent 695 DE genes, and IM single-agent 1118 DE genes. Of those, the GO + IM combination had 57 DE TKI-independent genes, GO single-agent 19 DE TKI-independent genes, CsA single-agent 9 DE TKI-independent genes and IM single-agent 17 DE TKI-independent genes (Table S4) (all DE genes available in Tables S5–S8). The number of DE TKI-independent genes was the one expected by chance in all conditions, as reported by hypergeometric distribution (p_combination_ = 0.82, p_IM_ = 0.72, p_GO_ = 0.71, p_CsA_ = 0.15), suggesting that none of the treatments specifically targets all the TKI-independent genes. This is expected by GO and CsA, as they are targeted therapies against only one TKI-independent gene each, *CD33* and *PPIF*, respectively. However, we expected the TKI-independent genes to be under-represented in the IM-treated cells.

Therefore, we next investigated why the TKI-independent genes were not under-represented after the IM treatment. This includes *CD33* and *ERG*, which were found to be TKI-independent genes also by qPCR. As *ERG* was upregulated after IM treatment, we hypothesised that IM enriched for LSC in culture, as previously reported [7]. Thus, we used xCell [39] to calculate the HSC/LSC enrichment score in all treatment conditions and compared them to the NDC as well as single agent GO and IM to the combination treatment. xCell allows for the estimation of cell type enrichment across different samples using gene signatures derived from well-annotated datasets, such as the Human Primary Cells Atlas [40]. Interestingly, the IM-treated cells showed significant enrichment in HSC/LSC compared to the NDC (FDR = 0.01) (Figure 5c, Table S9). As this enrichment was not detected in the TKID microarray dataset (Figure S9), the DE TKI-independent genes are likely DE due to the change in cell-type composition in the IM-treated cells compared to the NDC, similar to previous reports [7].

Following this, we investigated if any other cell type expression signatures were enriched in any of the conditions. We found that the GO + IM combination was borderline significantly enriched in megakaryocytes when compared with the NDC or any of the two drugs alone (FDR < 0.1) (Figure 5d, Table S10), suggesting that the combination treatment may promote HSC/LSC differentiation towards the megakaryocytic lineage. This difference was not detected in TKID (Figure S10).

Additionally, the gene expression changes (compared to NDC) for each condition were analysed for pathway enrichment using GSEA. We found that CsA upregulates the pathways related to cell death while it downregulates pathways associated with p53 (Figure 5e). This was expected as the described mechanism of CsA is the inhibition of PPIF, which is known to interact with p53, promoting necrosis, and with anti-apoptotic BCL2, preventing programmed cell death [15]. IM also showed an expected effect, with cell-cycle-related pathways being downregulated after IM treatment. GO showed an enrichment of the pathways involved in the DNA double-strand break response, which was expected because of the mechanism of action of calicheamicin. Interestingly, the GO-treated cells also showed an enrichment of the pathways involved in cell differentiation through RUNX1/2 and KIT, especially in combination with IM (Figure 5e and Figure S8). The results from the GSEA support the effects of the drugs observed experimentally and the proposed mechanisms of action of GO and CsA.

## 4. Discussion

The existence of a BCR-ABL1 TK-independent gene expression signature that allows CML LSC to persist after TKI treatment has been long hypothesised [2,3,4,9,10]. Indeed, different signalling pathways, such as the β-catenin [12] MYC and p53 pathways [13], have been shown to be unaffected by TKI treatment but are deregulated in CML LSC compared with their healthy HSC counterparts. In this study, we define a list of 227 genes (Table S3) that are differentially expressed in CML CD34^+^ cells but are not affected by TKI treatment using pre-existing microarray datasets. Despite the small sample size and the lack of in vivo post-TKI patient cells, this list suggests the existence of a transcriptomic signature in CML that is not affected by TKI and which is likely independent from the TK activity of BCR-ABL1 (although not necessarily BCR-ABL1 independent).

We have also shown that targeting the gene products from our list with existing compounds, such as GO and CsA (which target CD33 and PPIF, respectively), could open new avenues for the treatment of CML. GO is currently marketed for the treatment of AML, and it seems to have low long-term toxicity on healthy HSCs at its recommended dose [35]. This low HSC toxicity is supported by our experiments, as we observed an EC_50_ > 3000 ng/mL in non-CML CD34^+^ cells (897 ng/mL in combination with 2 µM IM). On the other hand, we have shown that GO is effective at reducing the number of CML CD34^+^ cells. Previous studies have shown that GO is effective against CML chronic phase (CML-CP) mononuclear cells in vitro [36], and it has been used with partial success when treating patients with TKI-resistant CML blast crisis [41,42,43]. Here, we show that GO could be used for the treatment of CML-CP as it seems to push CML CD34^+^ cells into the cell cycle (Figure 3e) and drives CML LSC into differentiation, as suggested by the sequencing results (Figure 5e). This might be induced by an increase in reactive oxygen species (ROS) [44]. However, GO induces DNA damage in the CML cells, so when the leukaemic clone is not eradicated, it might increase the risk of additional mutations that could lead to clonal evolution, treatment resistance or disease progression [25,45,46,47]. Thus, it might be unfavourable for patients with a good risk profile, and long-term follow-up is required to further evaluate this.

Interestingly, PPIF is a key regulator of autophagy [48] and ROS/ischemia-mediated necrosis through interaction with p53 [15]. Conversely, PPIF interacts with BCL2 to inhibit apoptosis, potentially through the inhibition of cytochrome C release [49]. Thus, PPIF is required for necrosis, but it also prevents apoptosis. High expression levels of *PPIF* might be explained by the increase in mitochondrial mass [50] and reduced necrosis-driven cell death by the reduced activity of p53 in CML LSC [13]. CsA has been used to block the interaction of PPIF with both p53 [15] and BCL2 [49], providing an anti-necrotic and pro-apoptotic effect. This is in line with our CFC results. While healthy HSC are more sensitive to CsA in the absence of IM (as they lack the pro-survival signalling from BCR-ABL1 TK), the combination of CsA and IM increases cell death in CML CFC. Although the inhibition of PPIF might be a potential therapy in CML, CsA is not specific, and its EC_50_ in our laboratory was not a clinically achievable concentration. Thus, more targeted compounds would need to be developed.

Previous reports have shown that CD26 (HUGO nomenclature: *DPP4*) [51] and CD93 [34] are expressed in CML cells with repopulation potential, suggesting that these markers could be used as biomarkers for CML LSC. Interestingly, CML CD26^+^ cells have been selectively targeted in vitro using an immunoliposome system loaded with venetoclax, showing that these proteins can also be used to therapeutically target subpopulations of leukaemic cells [52]. However, we did not find either of these markers to be TKI-independent in our bulk microarray and RNAseq experiments. As both CD26 and CD93 are markers for CML LSC, their gene expression after TKI treatment may change due to the enrichment for HSC-like cells we show in Figure 5c. Furthermore, the differences between the gene and protein expression are not uncommon: we previously reported similar expression levels of *CD93* between CD93^+^ and CD93^−^ CML LSC in a single-cell experiment [34]. Further investigation exploring both gene and protein expression (e.g., performing CITEseq) in patients treated with TKI may help refine a list of TKI-independent targets and/or biomarkers. 

## 5. Conclusions

In summary, we have confirmed the existence of a TKI-independent transcriptomic programme in CML CD34^+^ cells that is not present in healthy HSC. We have also demonstrated that TKI-independent genes can be used as a novel approach for target identification in CML LSC. However, this approach needs to be validated in cells from TKI-treated patients.

## Data Availability

The RNAseq data generated during this study can be accessed in GEO (GSE198576).

## Supplementary Materials

The following are available online at https://www.mdpi.com/article/10.3390/cancers14215253/s1, Figure S1: CD33 surface expression levels by flow cytometry in CML and non-CML CD34^+^ cells, Figure S2: Bar plot showing the ratio of CD33 expression levels on the cell surface between IM treated CD34+ cells and the no drug control (NDC) by flow cytometry, Figure S3: Representative plots of Annexin V (FITC) and DAPI (Pacific Blue) staining for a CML sample after treatment with GO, Figure S4: Representative plots of Annexin V (FITC) and DAPI (Pacific Blue) staining for a non-CML sample after treatment with GO, Figure S5: Summary of all the samples’ staining for Annexin V across the three GO/IM treatment regimens, Figure S6: Representative example of cell cycle analysis on a CML sample after treatment, Figure S7: Representative example of cell cycle analysis on a non-CML sample after treatment, Figure S8: GSEA enrichment scores of all the Reactome pathways significantly enriched in at least one treatment condition, Figure S9: xCell enrichments for HSC in TKID, Figure S10: xCell enrichments for megakaryocytes in TKID, Table S1: Summary of the CML primary patient samples used in this project, Table S2: Summary of the nCML samples used in this project, Table S3: List of TKIi genes with log2 fold-changes of datasets TKID and CMLD1, Table S4: Summary of the number of differentially expressed genes in the RNAseq experiment for each of the treatment conditions compared to the no drug control, Table S5: List of differentially expressed genes of the combination treatment 100 ng/mL GO + 2 µM IM compared to no drug control, Table S6: List of differentially expressed genes in GO (100 ng/mL) treated cells compared to no drug control, Table S7: List of differentially expressed genes in CsA (3 µM) treated cells compared to no drug control, Table S8: List of differentially expressed genes in 2 µM IM treated cells compared to no drug control, Table S9: Statistics of the pairwise t-tests performed for the enrichment of HSC using xCell on the bulk RNAseq. The shown t-tests are the only ones performed for the analysis, Table S10: Statistics of the pairwise t-tests performed for the enrichment of megakaryocytes using xCell on the bulk RNAseq. The shown t-tests are the only ones performed for the analysis.
