# Supplementary material for: Tyrosine Kinase Inhibitor Independent Gene Expression Signature in CML Offers New Targets for LSPC Eradication Therapy
Source: Cancers (Basel). 2022 Oct 26;14(21):5253. doi: 10.3390/cancers14215253 (PMC9655972; doi:10.3390/cancers14215253)
Supplement: Supplementary file 1 [file cancers-14-05253-s001.zip › cancers-1818536-supplementary.pdf]

Supplementary Figures

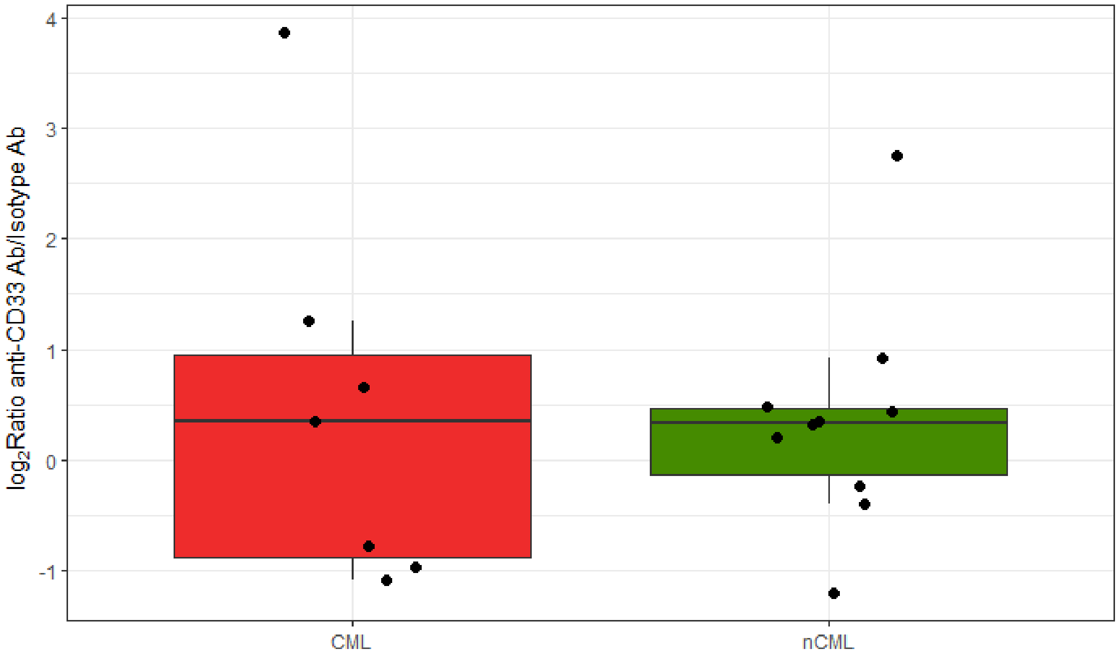

Figure S1. CD33 surface expression levels by flow cytometry in CML and non-CML CD34^{+} cells.

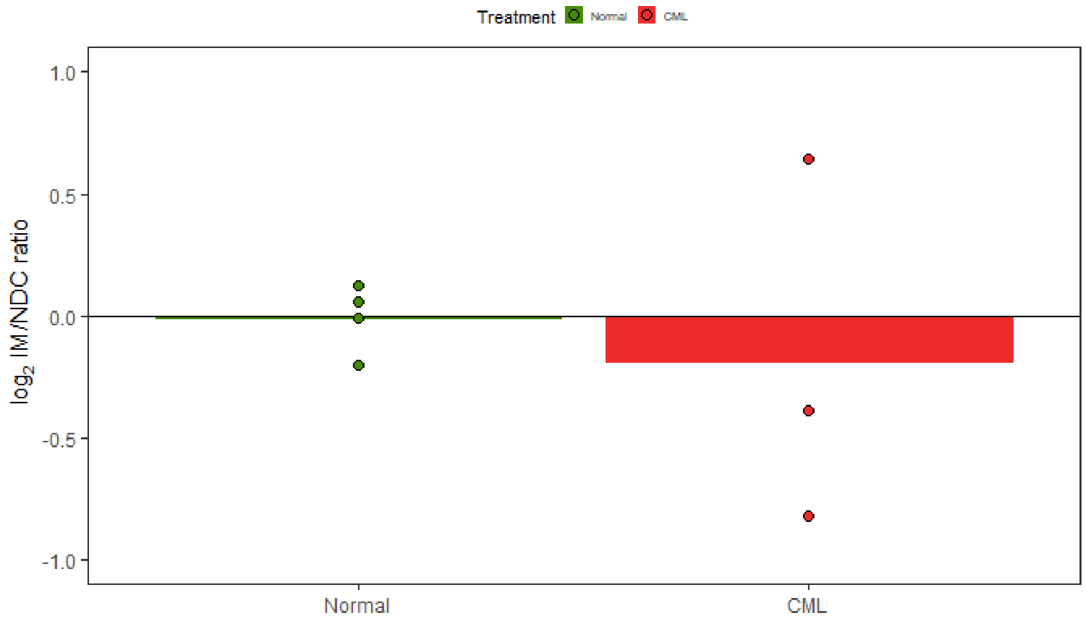

Figure S2. Bar plot showing the ratio of CD33 expression levels on the cell surface between IM treated CD34[+] cells and the no drug control (NDC) by flow cytometry.

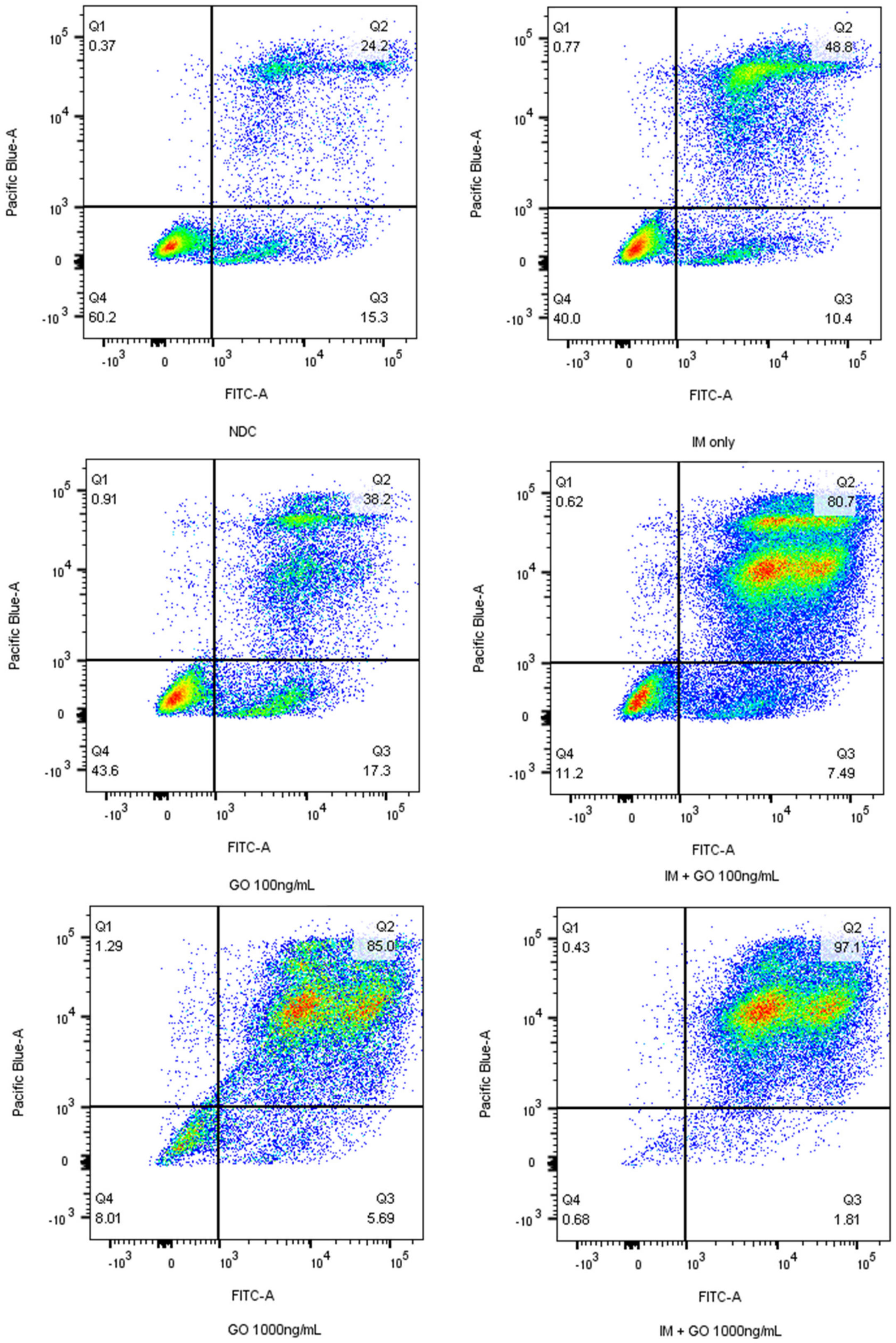

Figure S3. Representative plots of Annexin V (FITC) and DAPI (Pacific Blue) staining for a CML sample after treatment with GO.

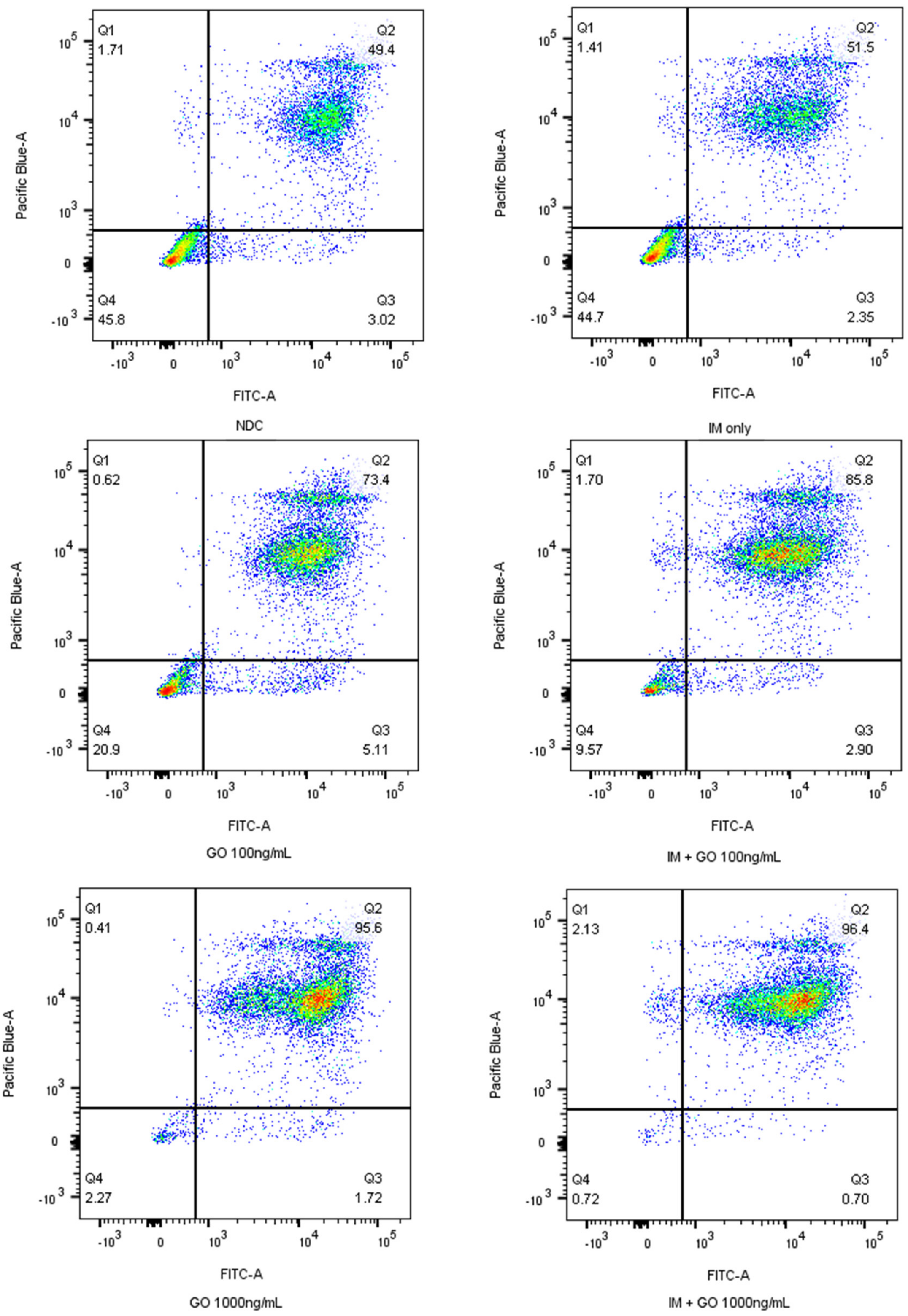

Figure S4. Representative plots of Annexin V (FITC) and DAPI (Pacific Blue) staining for a non-CML sample after treatment with GO.

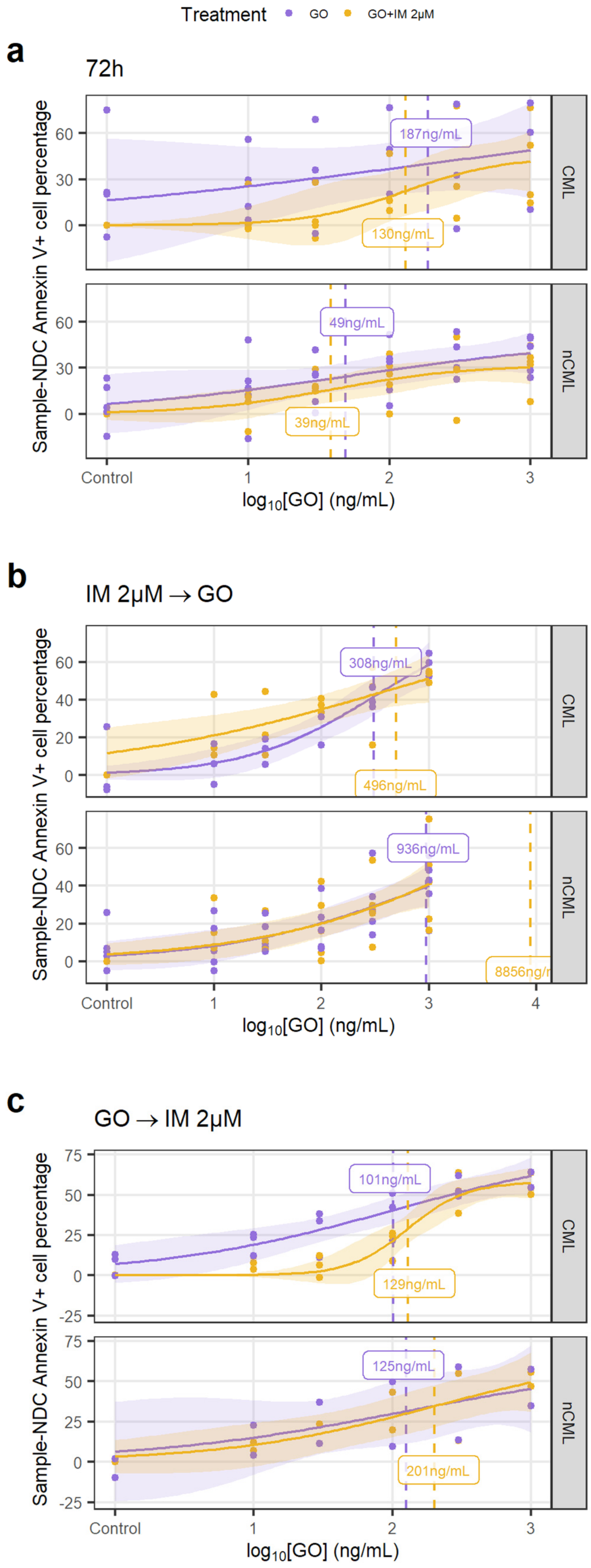

Figure S5. Summary of all the samples' staining for Annexin V across the three GO/IM treatment regimens: (a) GO_{72}, (b) GO_{IMGO} and (c) GO_{GOIM}.

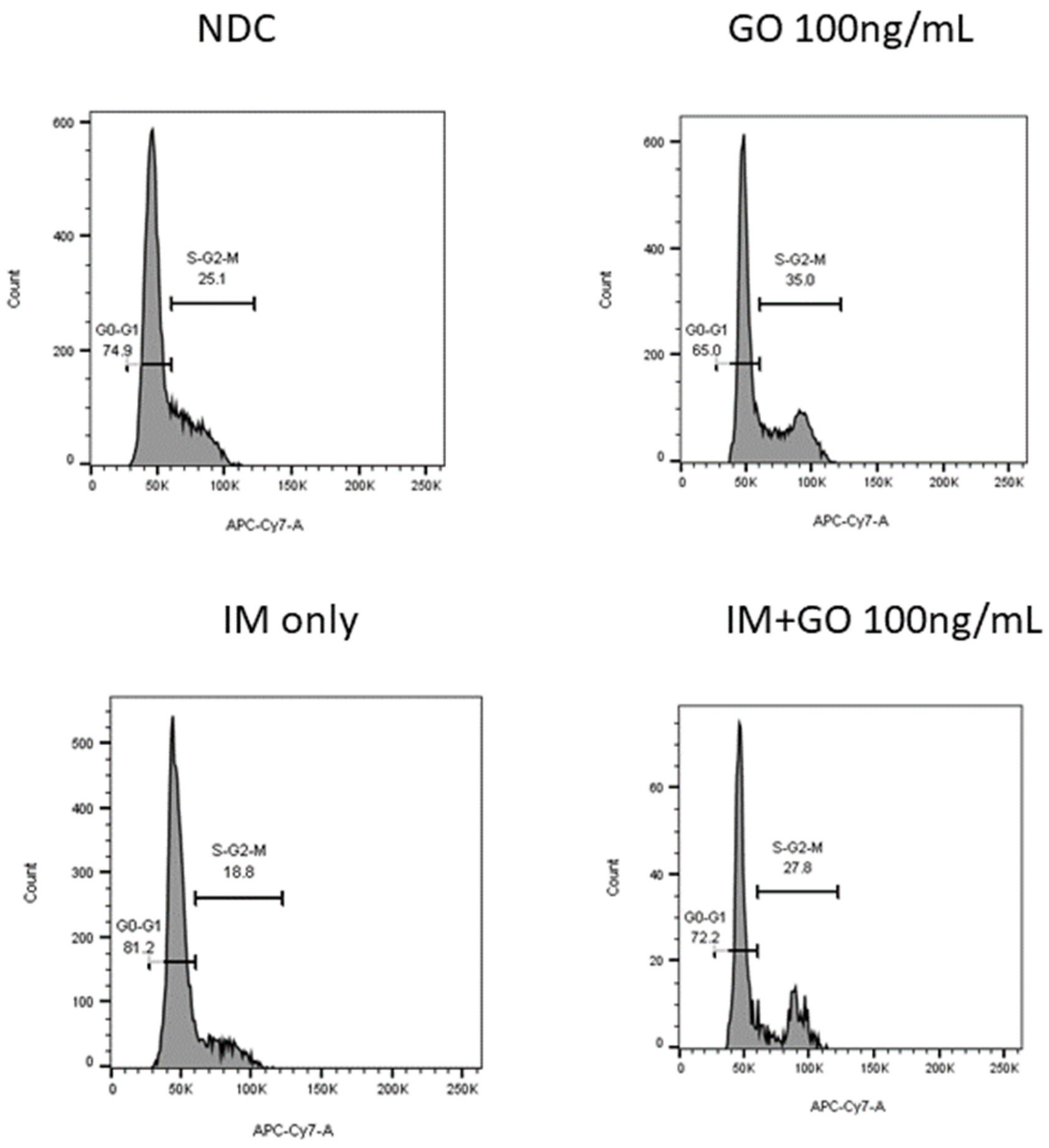

Figure S6. Representative example of cell cycle analysis on a CML sample after treatment.

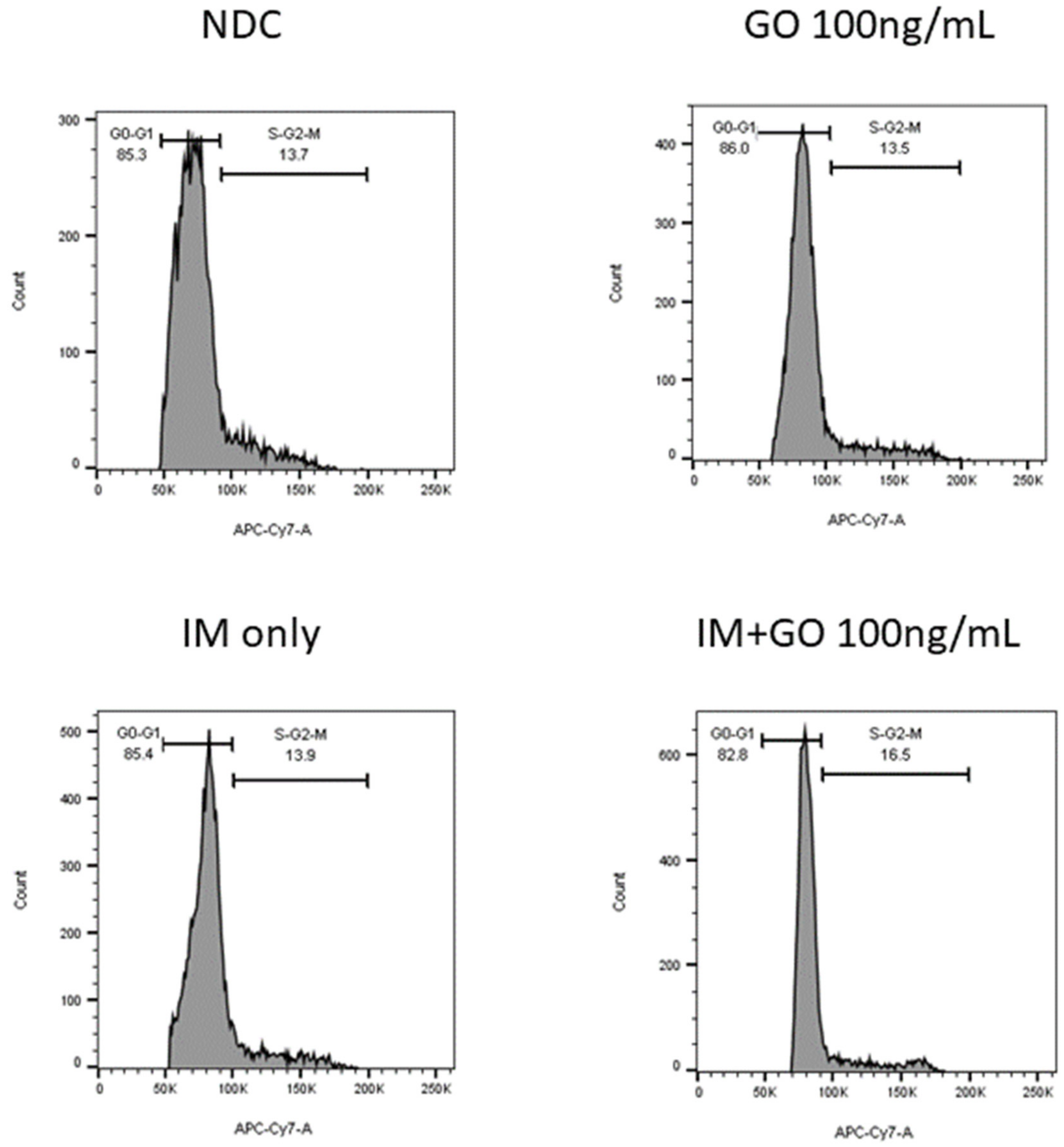

Figure S7. Representative example of cell cycle analysis on a non-CML sample after treatment.

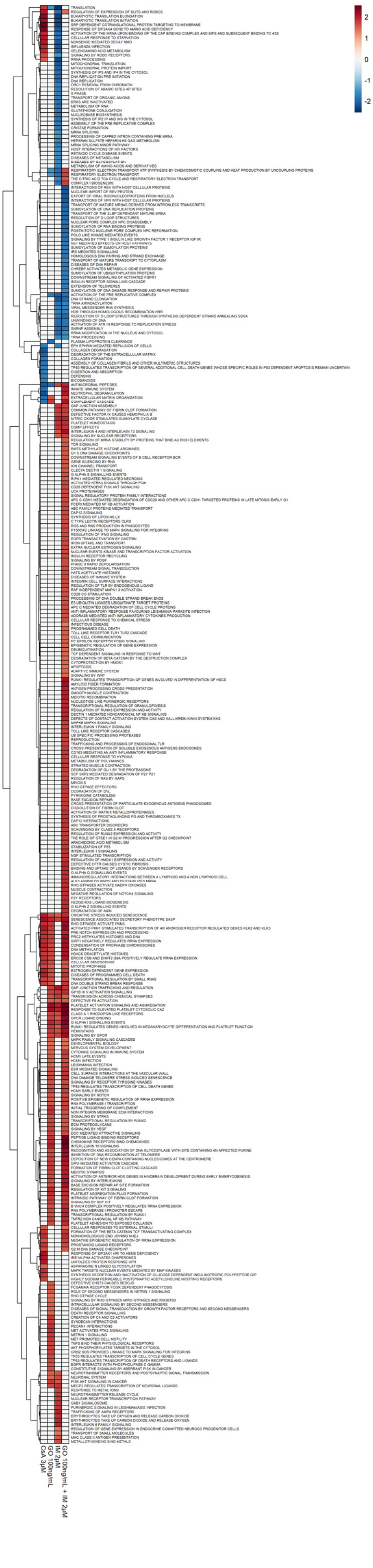
2
1
0
-1
-2
GO 100ng/mL + IM 2μM
IM 2μM
GO 100ng/mL
CsA 3μM

Figure S8. GSEA enrichment scores of all the Reactome pathways significantly enriched in at least one treatment condition. White squares represent non-significant pathways (independently of the enrichment score). Blue represents downregulated pathways while red represents upregulated pathways.

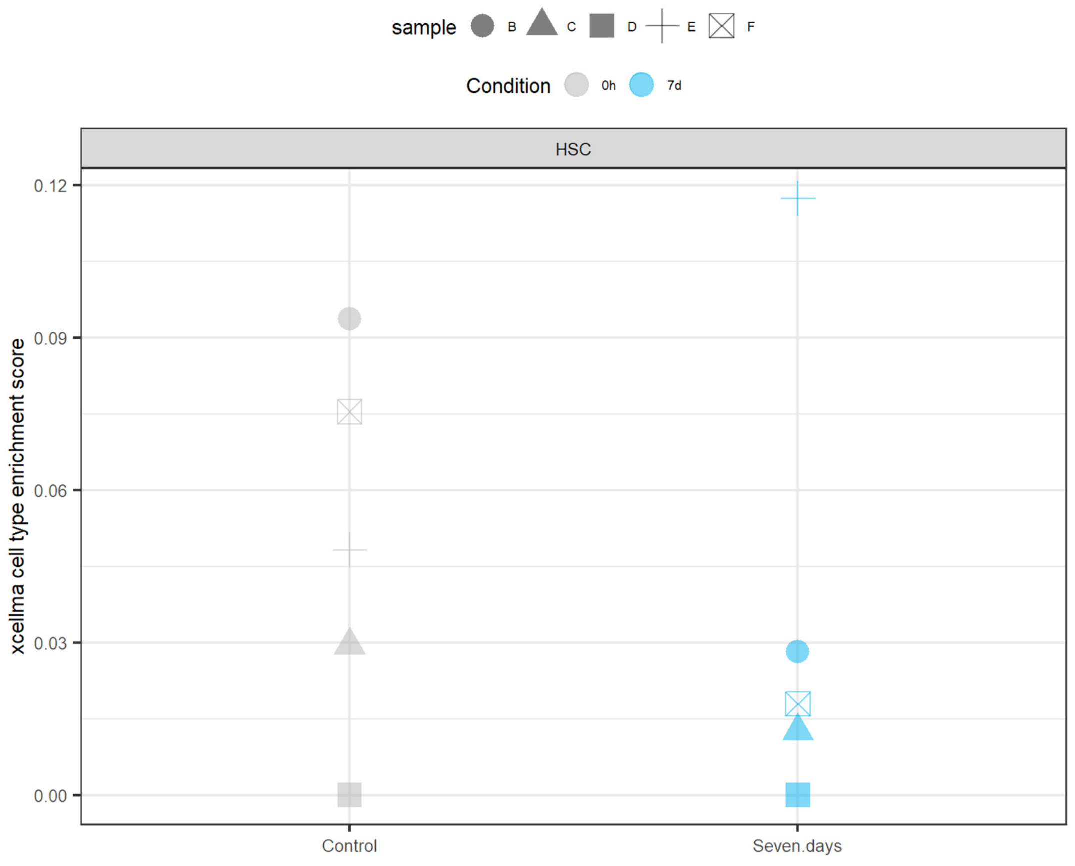

Figure S9. xCell enrichments for HSC in TKID. No significant difference was observed between the samples before and after treatment. Each shape represents an individual patient while each colour represents an individual time-point.

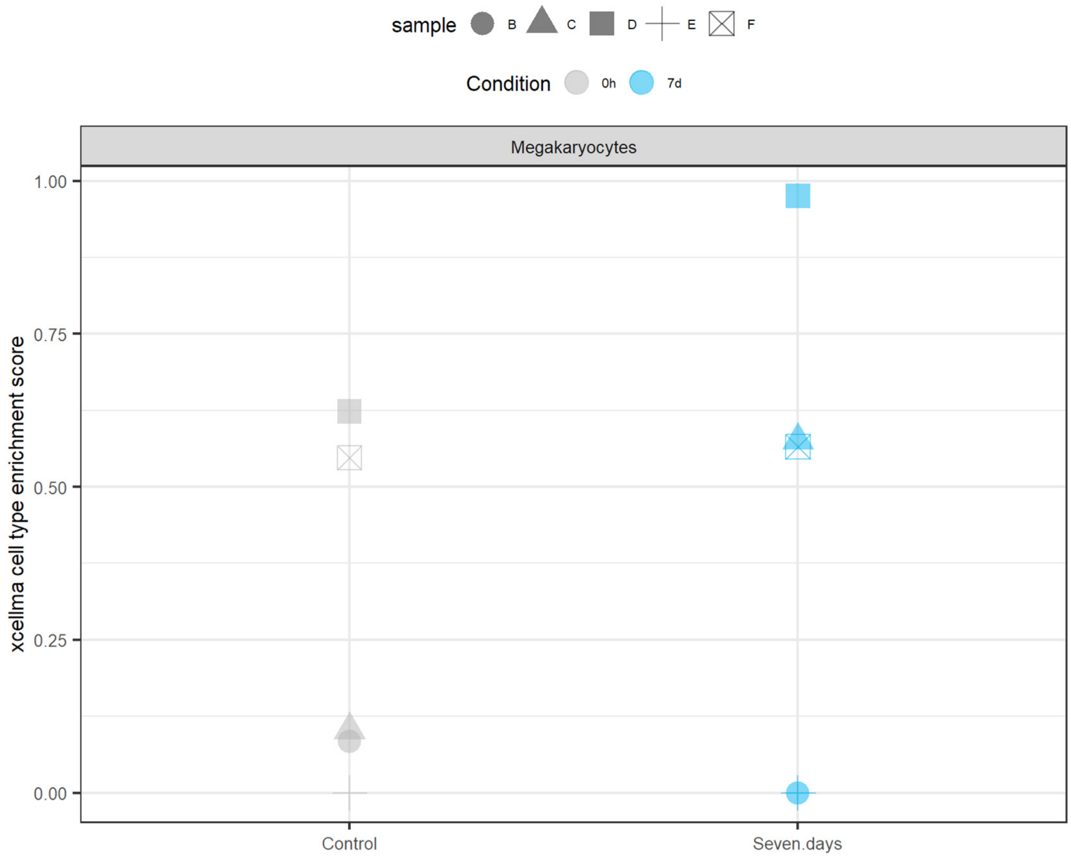

Figure S10. xCell enrichments for megakaryocytes in TKID. No significant difference was observed between the samples before and after treatment. Each shape represents an individual patient while each colour represents an individual time-point.

Supplementary tables

Table S1. Summary of the CML primary patient samples used in this project. The levels of BCR-ABL1 by qPCR refer to the 6 months checkpoint. n/a not available.

Table S2. Summary of the nCML samples used in this project. n/a not available.

Table S3. List of TKIi genes with $\log_2$ fold-changes of datasets TKID and CMLD1. The table also states if the genes were also analysed by qPCR.

Table S4. Summary of the number of differentially expressed genes in the RNAseq experiment for each of the treatment conditions compared to the no drug control. The table also states the number of TKIi genes differentially expressed for each condition.

Table S5. List of differentially expressed genes of the combination treatment 100ng/mL GO + 2µM IM compared to no drug control.

Table S6. List of differentially expressed genes in GO (100ng/mL) treated cells compared to no drug control.

Table S7. List of differentially expressed genes in CsA (3µM) treated cells compared to no drug control.

Table S8. List of differentially expressed genes in 2µM IM treated cells compared to no drug control.

Table S9. Statistics of the pairwise t-tests performed for the enrichment of HSC using xCell on the bulk RNAseq. The shown t-tests are the only ones performed for the analysis.

Table S10. Statistics of the pairwise t-tests performed for the enrichment of megakaryocytes using xCell on the bulk RNAseq. The shown t-tests are the only ones performed for the analysis.
